# New Pyridinium Type Poly(Ionic Liquids) as Membranes for CO_2_ Separation

**DOI:** 10.3390/polym10080912

**Published:** 2018-08-13

**Authors:** Aristofanis Vollas, Thanasis Chouliaras, Valadoula Deimede, Theophilos Ioannides, Joannis Kallitsis

**Affiliations:** 1 Department of Chemistry, University of Patras, GR-26504 Patras, Greece; aristofanis.vollas@gmail.com (A.V.); thxouliaras@yahoo.gr (T.C.); kallitsi@upatras.gr (J.K.); 2 Foundation for Research and Technology-Hellas, Institute of Chemical Engineering Sciences (FORTH/ICE-HT), GR-26504 Patras, Greece; theo@iceht.forth.gr

**Keywords:** gas separation, polymeric ionic liquids, pyridinium, PIL-IL composite membranes

## Abstract

New pyridinium based PILs have been prepared by modification of their precursors based on high molecular weight aromatic polyethers bearing main chain pyridine units. The proposed methodology involves the conversion of the precursors to their ionic analogues via *N*-methylation reaction, followed by anion exchange methathesis reaction to result in PILs with the desirable anions (tetrafluoroborate and bis(trifluoromethylsulfonyl)imide). These PILs show excellent thermal stability, excellent mechanical properties, and most importantly can form very thin, free standing films with minimum thickness of 3 μm. As expected, the PIL containing the TFSI^−^ anion showed improved CO_2_ and CH_4_ permeabilities compared to its analogue containing the BF_4_^−^. PIL-IL composites membranes have also been prepared using the same PIL and different percentages of pyridinium based IL where it was shown that the membrane with the highest IL weight percentage (45 wt %) showed the highest CO_2_ permeability (11.8 Barrer) and a high CO_2_/CH_4_ ideal selectivity of 35 at room temperature.

## 1. Introduction

Increasing carbon dioxide (CO_2_) emissions in the environment have contributed to global warming and climate changes [1], which are issues of paramount importance. As fossil-fuel power plants are currently the largest point sources of anthropogenic CO_2_ emissions, the efficient and economical removal of CO_2_ from power plant flue gas streams (CO_2_/N_2_) constitutes a key technical, financial and environmental challenge [2]. Removal of CO_2_ form natural gas and biogas can be also viewed in a similar context. Various separation technologies are being developed for the aforementioned processes including absorption with amines, pressure swing adsorption using porous solids and cryogenic separation [3]. Membrane gas separation technology based on polymeric materials is also one of the most widely studied and fast growing separation methods. It offers several advantages over the abovementioned technologies including low energy consumption and operating costs, easy scale up and adaptation into the existing processes [4,5].

Polymeric ionic liquids or poly(ionic liquid)s (PILs) as CO_2_ separation membrane materials have become an emerging field with great potential in recent years, offering the advantage of better stability compared to supported ionic liquid membranes (SILMs) while preserving most of their properties associated with their ionic liquid (IL) character [6,7,8]. PILs combine the highly tunable nature of ILs, with the improved mechanical stability of the polymers, paving the way to a new generation of functional materials with plethora of applications in diverse fields such as energy (lithium ion batteries, solar cells, fuel cells), environment (water treatment, ion exchange resins), catalysis, microelectronics, bio related applications [9,10,11,12,13]. The mechanism governing gas transport through PIL dense membranes is the one of solution-diffusion [14], thus, CO_2_ solubility is one of the key factors determining the separation performance of PIL membranes. In addition, both the polymer backbone and the type of ions affect the CO_2_ sorption capacity [12]. For PILs several studies demonstrate that the chemical composition of both cationic pendants and anions influence the CO_2_ sorption [9,15,16,17,18].

One of the identified critical challenges for developing polymeric membranes for gas separation is to deal with the “trade off” behavior [19,20]: highly permeable membranes lack selectivity and vice versa. Thus, PILs with varying configurations have been proposed including neat PILs membranes, PIL/IL composite membranes, PIL/IL mixed matrix-based membranes. Neat PILs composed of alkyl imidazolium cations tethered to a polystyrene, polyvinyl or polyacrylate backbone, with NTf_2_^−^ anions as counter ions are the most well known PILs based membranes that were synthesized via free radical polymerization [17,21,22,23,24]. Alternative IL cation moieties such as ammonium [17,25,26,27], pyridinium [17], pyrrolidiniuum [28,29] based PILs membranes for CO_2_ separation have also been developed. However, the majority of these PILs show poor mechanical stability due to the combination of low molecular weights and low glass transition temperature (*T*_g_) [30], and therefore could not be formed into robust membranes.

An alternative solution to overcome this problem is the development of PILs based on rigid and fully aromatic backbones with cationic moieties in the main chain or as side chains via condensation reaction. Synthesis of these PILs is usually conducted either by condensation of the respective ionic monomers or via polymer modification. The first route involves complex preparation and purification steps, while the second one enables the facile conversion of commercially available polymers with high molecular weights to their corresponding PILs. Very recently, PILs based on polybenzimidazole (PBI) [31,32,33], polyurethanes [34], polyimides (PI) bearing benzimidazole or quinuclidine units have been also proposed [30]. Aromatic polyethers are generally characterized by excellent mechanical strength as well as chemical and thermal stability. Due to these advantageous characteristics, aromatic polyethers bearing main chain pyridine units can be employed as precursors for the preparation of CO_2_ gas separation membranes. Aromatic polyethers bearing pyridine units in the main chain synthesized via polycondensation [35,36,37,38] and used as high temperature polymer electrolytes for Proton Exchange Membrane Fuel Cells (PEMFC) as well as their respective N-methyl pyridinium derivatives [39] used in alkaline electrolyte Fuel Cells (FCs) have already been reported by our group.

This work is focused on the conversion of aromatic polyethers bearing main chain pyridine groups to their novel pyridinium based PILs that can form very thin, free standing films with thickness ranging from 3 to 25 μm via a conventional solution casting method. The effect of the counter anion on film forming ability, thermal stability, as well as on the gas permeation properties was investigated. In parallel, and in order to further improve the CO_2_ permeability, PIL/IL composite membranes containing the TFSI^−^ anion (in both IL and PIL) were also prepared. Different amounts (30, 40, 45 wt %) of a pyridinium based IL were blended with PILs (PIL and IL have the same cation) to evaluate the effect of the free IL on the CO_2_ permeability and selectivity as well as on film forming ability. Blend membranes were also fabricated by blending different alkyl substituted imidazolium based ILs with PILs to study the IL cation influence on gas separation properties.

## 2. Materials and Methods

### 2.1. Materials

Aromatic polyethers bearing main chain pyridine units (Py-APE, *M*_n_ 60,000 g/mol), were synthesized according to a previously reported procedure [38]. Dimethyl Sulfate (DMSA, ≥99%), *N*,*N*-Dimethylacetamide (DMAc, anhydrous, 99.8%), *N*,*N*-Dimethylformamide (DMF, anhydrous, 99.8%), Toluene (99.7%), K_2_CO_3_ (99%+), Lithium bis(trifluoromethylsulfonyl)imide (LiTFSI, ≥99%), 1-Butyl-4 methyl pyridinium chloride ([bmpy][Cl], 97%) and dichloromethane (DCM, 99.9%) were purchased from Aldrich (St. Louis, MO, USA). The ionic liquids 1-hexyl-3-methyl-imidazolium (trifluoromethylsulfonyl) imide ([c6mim][TFSI], >98%) and 1-dodecyl-3-methyl-imidazolium (trifluoromethylsulfonyl) imide ([c12mim][TFSI], 99%) were supplied from IoLitech GmbH (Heilbronn, Germany). The exchange of chloride anion of 1-Butyl-4 methyl pyridinium chloride ([bmpy][Cl]) to the TFSI^−^ anion was performed following a previous work [32]. Sodium tetrafluoroborate (NaBF_4_, 97%) was supplied by Alfa Aesar (Karlsruhe, Germany). All other solvents were used as received. Before use, ionic liquids were dried under vacuum (10^−3^ kPa) at a moderate temperature of 45 °C overnight in order to reduce the water and other volatile substances. Single gases, He, CO_2_, CH_4_, N_2_ were supplied by Air Liquid with at least 99.99% purity.

### 2.2. Synthesis of PILs

**Scheme 1:**
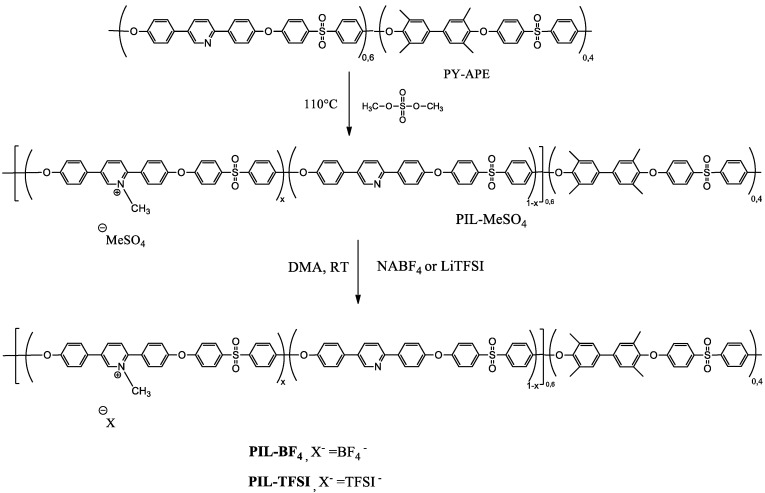
Synthetic route for the synthesis of pyridinium based PILs.

PILs have been prepared via conversion of precursor polymers based on high molecular weight aromatic polyethers bearing main chain pyridine units to their ionic analogues via *N*-methylation reaction [39], followed by anion exchange metathesis reaction using the desirable salts NaBF_4_ and LITFSI (Scheme 1).

#### 2.2.1. Synthesis of Precursor Py-APE

The synthesis of precursor aromatic polyethers bearing main chain pyridine units (Py-APE) was reported earlier by our group [38]. Polycondensation reaction of the tetramethyl biphenyl diol with 2,5 diphenyl substituted pyridine diol and diphenyl sulfone difluoride took place at 160 °C for 24 and 6 h at 180 °C using DMF/Toluene as solvents and K_2_CO_3_ as base. The viscous solution was precipitated in an excess of MeOH/H_2_O 2/1, washed with water and hexane and dried at 80 °C under vacuum. The 60/40 molar ratio was chosen since this ratio combines the high pyridine content with solubility, thus resulting in Py-APE copolymers with high molecular weights (*M*_n_ = 60,000 g/mol, *M*_w_ = 125,000 g/mol, PDI = 2.1).

#### 2.2.2. *N*-Quaternization of Py-APE

In a 100 mL flask, 1 g of Py-APE and 40 mL of dimethylsulfate was added slowly over a period of 10 min while stirring, under argon atmosphere. The temperature was gradually raised to 110 °C and the reaction mixture was left stirring for 24 h. The deep yellow reaction mixture was cooled down to ambient temperature and was precipitated in an excess of deionized water, filtered and washed with water until neutral pH. The obtained polymer was dried in vacuum oven at 60 °C for 2 days and stored in a desiccator until use.

#### 2.2.3. MeSO_4_^−^ Exchange of *N*-Quaternized Py-APE

Typically, in a 50 mL flask, 1 g of *N*-quaternized Py-APE was dissolved in 15 mL of DMAc. After the complete dissolution, 5 eq (2.63 g) of LiTFSI was added while stirring. During the addition of the LITFSI salt the formed LiMeSO_4_ salt as well as the anion exchange polymer remained dissolved in DMA. The reaction mixture left stirring for 24 h at room temperature in order to ensure the complete exchange by TFSI^−^ anions. The obtained PIL-TFSI was precipitated in water, filtered and thoroughly washed with water and methanol in order to remove the excess of LiTf_2_N and LiMeSO_4_. It was further purified by dissolving in DMA (5% *w/v*), reprecipitation in water and drying at 60 °C in a vacuum oven for 24 h. The same procedure was followed for BF_4_^−^ using NaBF_4_ salt.

### 2.3. Membrane Preparation

The neat PIL membranes were prepared by the solution casting method on a flat glass surface using 3% (*w/v*) PIL solution in DMAc at 80 °C for 24 h under dry conditions. After solvent evaporation, the formed membrane was peeled off and dried in a vacuum oven at 80 °C (usually 2 days) until constant weight was achieved.

**Scheme 2:**
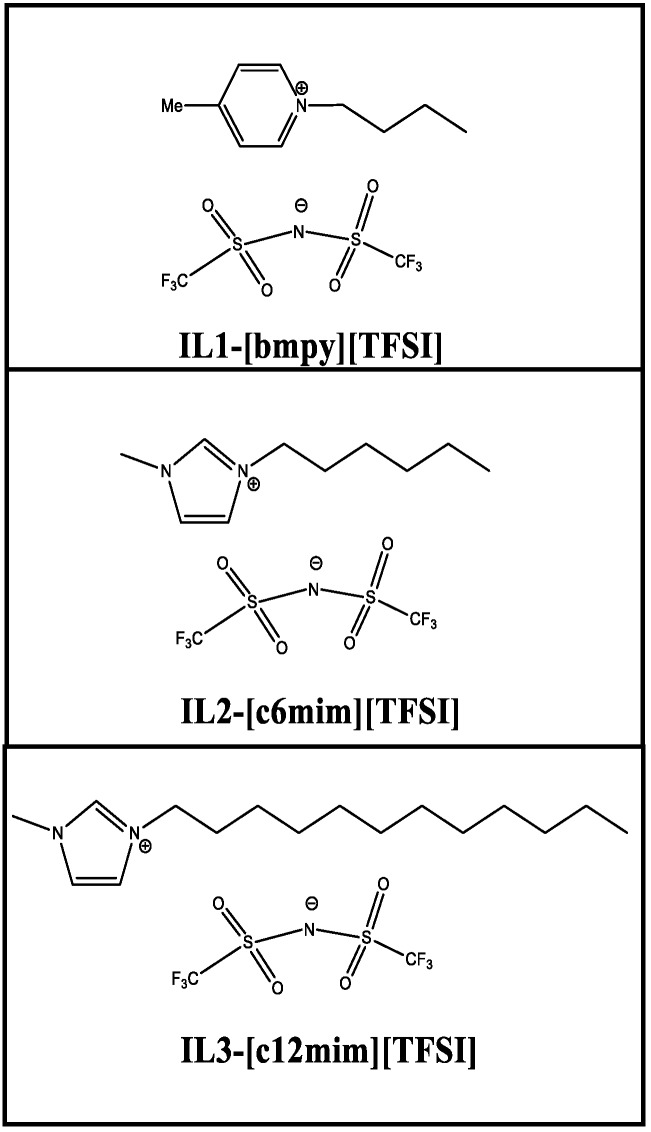
Chemical structures of the ionic liquids used to prepare PIL-IL composite membranes.

The PIL-IL membranes were also prepared using the solution casting method. In specific, different amounts (wt %) of ILs ([bmpy][TFSI], [c6mim][TFSI] and [c12mim][TFSI]) were added in a 5% (*w/v*) PIL-TFSI solution (DMAc is the solvent) in order to obtain solutions containing different weight percentages of free IL to the polymer matrix (Table 1 and Scheme 2). The solutions were magnetically stirred for 5 h at room temperature until both components were completely dissolved and a homogeneous solution was obtained. Then, the solutions were poured into a flat glass surface and left for slow evaporation of the solvent at 80 °C for 2 days. Finally, all the membranes were peeled off and dried in a vacuum oven at 80 °C (usually 2 days) until constant weight was achieved.

The thickness of PIL membranes (3–25 μm) as well as PIL-IL membranes (60–90 μm) was measured using Scanning Electron Microscopy (SEM) microscopy (Zeiss, Oberkochen, Germany), where average thickness was calculated from six measurements taken at different locations of each membrane sample. It should be mentioned that leaching of the free IL may take place more easily in the case of thin PIL-IL membranes (25–30 μm) with high IL content. Thus, thicker PIL-IL membranes were prepared compared to neat PIL membranes in order to avoid the undesirable possible leaching of the free IL.

### 2.4. Characterization

^1^H NMR spectra were obtained on an Advance DPX 400 MHz spectrometer (Bruker, Karlsruhe,, Germany). The samples were dissolved either in deuterated chloroform (CDCl_3_) or dimethylsulfoxide (DMSO-*d*_6_) with tetramethylsilane (TMS) used as internal standard. Thermogravimetric analysis (TGA) was performed using a Labsys TG (Setaram Instrumentation, Caluire-et-Cuire, France). The samples were heated at 20 °C min^−1^ to 800 °C under nitrogen atmosphere. ATR-IR spectra were recorded on Platinum ATR spectrometer (Bruker, Ettlingen, Germany). The surface and the cross-sectional morphology of the membranes were studied by SEM using a LEO Supra 35VP microscope (Zeiss, Oberkochen, Germany). The specimens for the cross-section study were prepared by fracturing the membranes in liquid nitrogen. Differential Scanning Calorimetry analyses were performed on a DSC instrument from Perkin Elmer (DSC Q100, Waltham, MA, USA) over a temperature range from −100 to 300 °C (including heating and cooling cycles) under nitrogen flow at a scan rate of 10 °C/min. The first heating run was conducted in order to remove the thermal history and traces of absorbed water and residual solvents from the samples. The glass transition temperatures were recorded in the second heating run.

### 2.5. Gas Permeation Measurements

CO_2_ and CH_4_ permeation experiments (single-gas and in 50/50 vol % CO_2_/CH_4_ mixtures) were performed at room temperature employing the Wicke-Kallenbach method [40]. The measurement cell accommodated membranes in the shape of circular discs with diameter of 5 cm. Helium at a flow of 20 cm^3^ min^−1^ was employed as the purge gas in the permeate side which was maintained at a pressure of 1 atm. A gas chromatograph (GC-2014, Shimadzu, Kyoto, Japan) equipped with thermal conductivity and flame ionization detectors and Carboxen and Porapak Q columns was used for analysis of CO_2_ and CH_4_ concentration in the helium permeate stream.

Gas transport through a homogeneous dense membrane follows a solution-diffusion mass transfer mechanism [14]. The permeability (*P*) was determined using Equation (1) [41]:

(1) $$P=\frac{J}{\Delta p}l$$

where *P* is the permeability coefficient expressed in Barrer and Δ*p* = *p*_1_ − *p*_2_, *p*_1_ and *p*_2_ are the feed and permeate side partial pressures of the measured gas (cm Hg), respectively. *J* is the steady-state gas flux (cm^3^/cm^2^ s) and *l* is the membrane thickness (cm).

The permeation measurements were repeated with 3 different membranes prepared under the same conditions. According to the solution-diffusion model, the permeability of a gas (*P*) can be also expressed as the product of gas diffusivity (*D*) and solubility (*S*) across the membrane Equation (2):

(2) P=D×S

The ideal selectivity (*α*_*i*/*j*_), also known as permselectivity, was determined using the following equation, by dividing the permeability of the most permeable gas *i* (*P*_*i*_) by the permeability of the least permeable gas *j* (*P*_*j*_):

(3) aij=PiPj=(DiDj)×(SiSj)

The separation factor, (SF), for CO_2_/CH_4_ mixtures was determined from the ratio of the concentrations of the more permeable gas species *i* and the less permeable gas species *j* in the permeate divided by the ratio of the same gases *i* and *j* in the feed stream from the following expression:

(4) $$SF=\frac{y_{i}/y_{j}}{x_{i}/x_{j}}$$

where *y*_*i*_ and *y*_*j*_ are the mole fractions of gas components *i* and *j* in the permeate and *x*_*i*_ and *x*_*j*_ are the fractions of components *i* and *j* in the feed. SF is also referred as mixed gas selectivity in the text.

At least 3 membrane samples of each type were prepared and used for the gas separation measurements in order to provide the corresponding error bars.

## 3. Results and Discussion

### 3.1. Synthesis and Characterization of Pyridinium Based PILs

**Figure 1:**
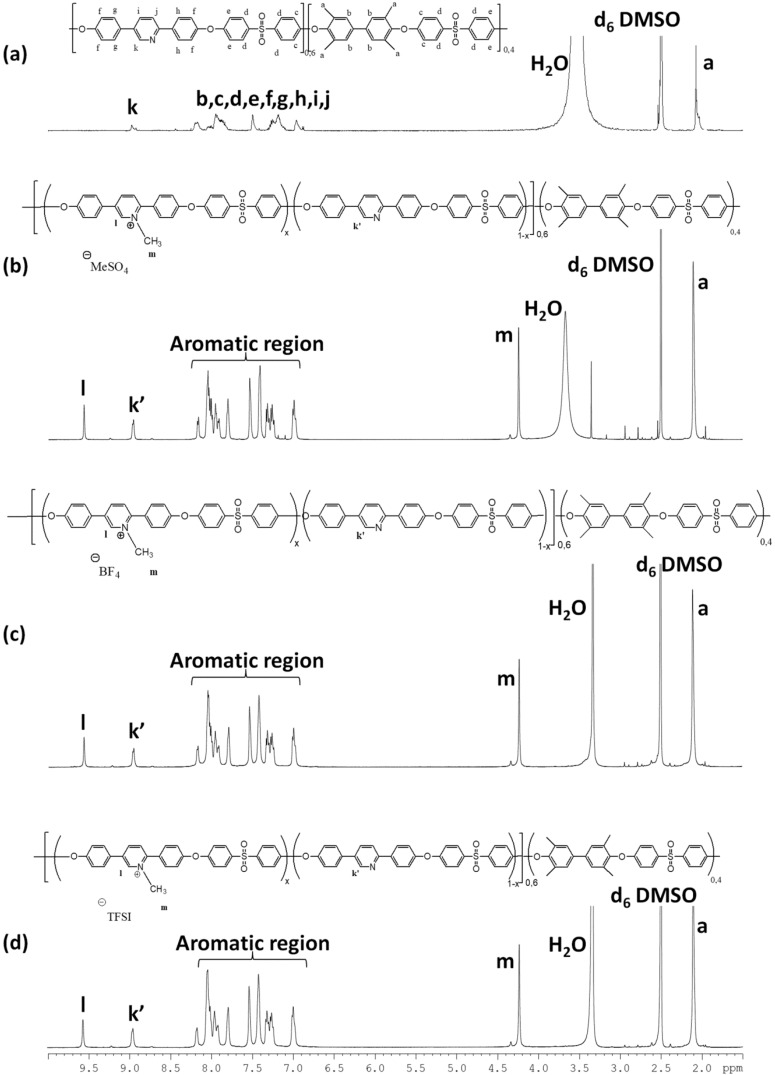
^1^H NMR spectra (DMSO-*d*_6_) of precursor polymer Py-APE (**a**) and its corresponding PIL with counter anions (**b**) MeSO_4_^−^, (**c**) BF_4_^−^ and (**d**) TFSI^−^.

Novel pyridinium based PILs have been synthesized via modification of the precursor aromatic polyether copolymers bearing main chain pyridine units. Different alkyl and aryl groups (e.g., ethyl bromide, benzyl bromide) were used as reagents for performing the *N*-quaternization reaction, however, the substitution reaction of pyridine was not successful. Thus, a more reactive reagent—dimethyl sulfate (with the smallest sized methyl group)—was chosen to be used as solvent and methylating agent simultaneously for the quaternization reaction [39]. The successful *N*-quaternization reaction resulted in high purity PILs (≥98%) as confirmed by ^1^H NMR spectroscopy. In Figure 1 the ^1^H NMR spectra of precursor copolymer Py-APE and the corresponding *N*-methyl pyridinium PIL containing the methyl sulfate anion are illustrated (Figure 1b). It is evident that after treatment with dimethyl sulfate, two new peaks appear, the first at 9.6 ppm and the second at 4.2 ppm which correspond to the aromatic proton **l** close to the pyridinium, and to the methyl group **m** directly attached to pyridinium, respectively, thus confirming the successful *N*-quaternization reaction. However, the conversion of pyridine units of the precursor to their pyridinium analogues is not complete. Consequently, the synthesized PILs contain both pyridine and pyridinium units, as evidenced by the presence of the aromatic proton **k’** close to the nitrogen of pyridine units which is located at 9.0 ppm. In order to achieve high *N*-quaternization degrees, different reaction times (24, 48 and 72 h) were followed. However, it was found that the same quaternization degree was obtained for all experiments (50%). The degree of *N*-substitution was estimated by comparing the aromatic protons at 9.0 and 9.6 ppm attributed to the **k’** proton close to the nitrogen of pyridine units and the corresponding **l** of the methyl-substituted pyridinium PIL, respectively (Figure 1a,b). The relatively moderate degree of quaternization obtained could be attributed to the steric hindrance of the pyridine units to accommodate the *N*-methyl group.

Due to insolubility of PIL containing MeSO_4_^−^ as counter anion in water, the ion exchange metathesis reactions using LITFSI and NaBF_4_ salts were carried out in DMAc. A larger excess of LITFSI or NaBF_4_ salt was necessary to achieve complete anion exchange in contrast to the synthesis of TFSI-based ILs [42]. Further evidence for the successful exchange to TFSI^−^ and BF_4_^−^ anions was collected by ^19^FNMR spectroscopy (ESI, Figures S1 and S2, respectively). In specific, a peak at −73.9 ppm appears which is assigned to the TFSI^−^ anion (Figure S1), while the peak located at −143.5 ppm is attributed to the BF_4_^−^ group (Figure S2).

**Figure 2:**
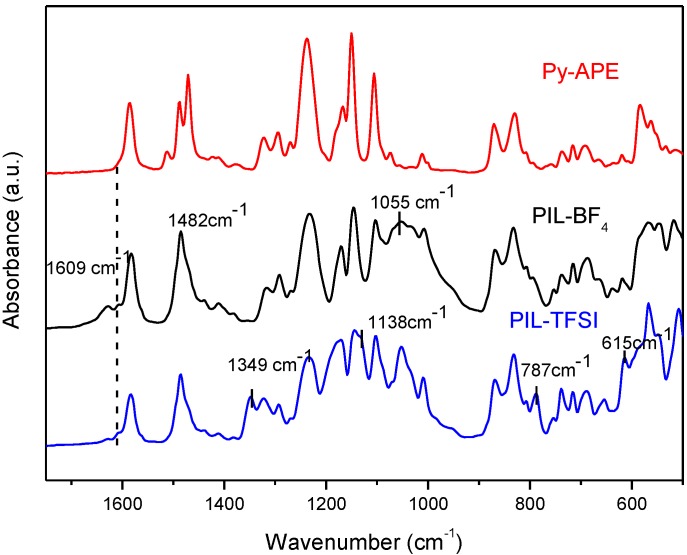
FT-IR spectra of precursor polymer Py-APE and its corresponding PILs with different counter anions.

The chemical structure of the synthesized PILs was verified through FTIR spectroscopy. A comparison of the PILs spectra with the precursor polymer are given in Figure 2. The precursor sample has two peaks located at 1471 and 1487 cm^−1^ corresponding to the C=C/C=N of pyridine ring. After modification, a broader, much stronger peak centered at 1482 cm^−1^ compared to the corresponding of precursor polymer and a shoulder located at 1468 cm^−1^ are observed. As 50% of the pyridine units were converted to pyridinium, the intensity of the sharp peak at 1471 cm^−1^ of the precursor was much reduced and became a shoulder in the pyridinium analogue. Moreover, a new peak appears in PILs spectra after methylation at 1609 cm^−1^ attributed to the stretching vibration of the newly formed C–N bond [39]. Thus, this evidence confirms the successful *N*-quaternization reaction. Moreover, the successful incorporation of the BF_4_^−^ and TFSI^−^ counter anions via anion exchange reactions was supported by the appearance of new peaks. In specific, in PILs possessing the BF_4_^−^ anion, a new band appeared at ~1055 cm^−1^ which is attributed to the B–F stretching vibration [43]. In the case of PIL possessing the Tf_2_N^−^ anion, bands at ~1349 cm^−1^ and in the range of 600–615 cm^−1^ are assigned to the asymmetric stretching and bending vibrations of SO_2_, respectively. In addition, the band at 787 cm^−1^ is attributed to the combination of the C–S and S–N stretching bands [44], while the bands in the range of 1138–1237 cm^−1^ correspond to the symmetric stretching vibrations of the C–F bond [45].

**Figure 3:**
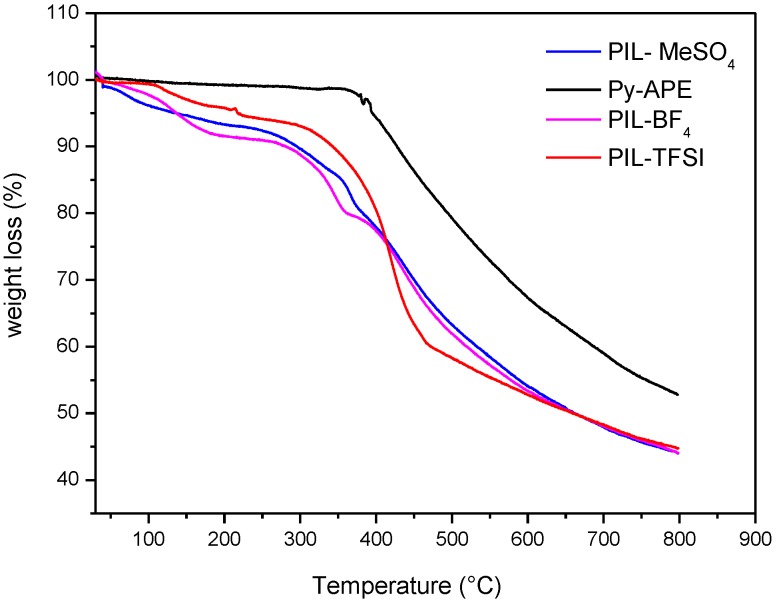
TGA curves of precursor polymer Py-APE and its corresponding PILs with different counter anions.

The thermogravimetric curves of precursor polymer Py-APE and its corresponding PILs with different counter anions are given in Figure 3. A closer inspection of TGA curves reveals that PIL-MeSO_4_ and PIL-BF_4_ lost 5% of their initial weight at temperatures up to 136 °C, while PIL-TFSI lost only 2.5%. This weight loss is attributed to absorbed water since MeSO_4_ and BF_4_ anions are more hydrophilic compared to the corresponding PIL containing the TFSI anion. Up to 210 °C, PIL-MeSO_4_ has a total weight loss of 7%, PIL-BF_4_ has 9% and PIL-TFSI only 4%. As these PILs were measured in film form and were prepared by solution casting using DMAc as solvent, the additional weight loss corresponds to residual DMAc solvent. Moreover, it is evident that all PILs exhibit multi step degradation in contrast to precursor polymer Py-APE which has only a single weight loss step, with an onset degradation temperature (*T*_ODT_) at 340 °C. The multi step degradation behavior corresponds to the ionic nature of PILs [32]. In addition, the precursor polymer shows improved thermal stability compared to their corresponding PILs. This suggests that the induced ionic character of PILs results in lower thermal stability. The deterioration of thermal stability of quaternized polymers compared to their unquaternized counterparts was also reported by others [31,46]. The comparison of the PILs with BF_4_^−^ and TFSI^−^ as counter anions, showed that the latter possess increased thermal stability (*T*_ODT_ ~285 °C) which could be attributed to its reduced basicity. In specific, it is known that the thermal stability of ionic liquids increases with decreasing anion basicity [47].

**Figure 4:**
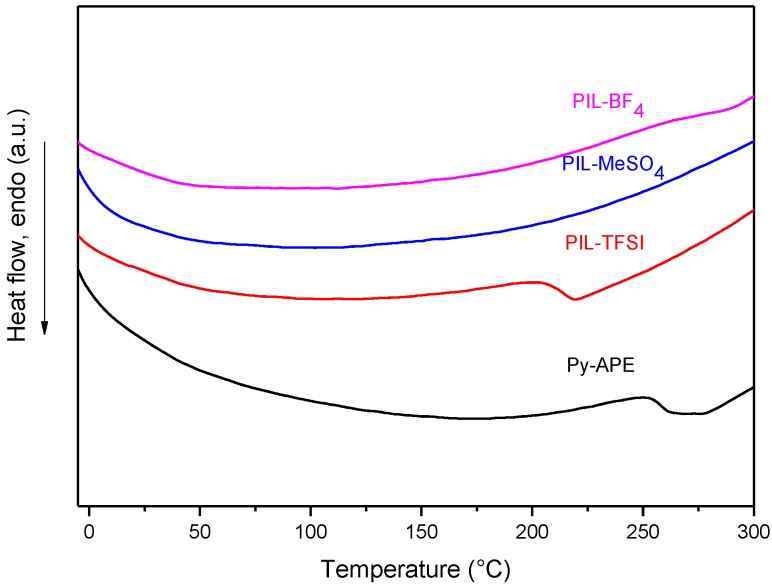
DSC curves of precursor copolymer and its corresponding PILs with different counter anions.

Only the glass transition temperatures of precursor polymer and PIL containing the TFSI^−^ anion could be detected in the DSC thermograms (Figure 4). Their glassy character was confirmed by their high *T*_g_ values. The conversion of the precursor polymer to its PIL analogue (PIL-TFSI^−^) led to a notable *T*_g_ decrease (from 258 to 214 °C, respectively). This behavior could be due to the incorporation of cation substituent as well as to the bulky TFSI^−^ anion which both disrupt the close polymer packing, thus leading to a lower *T*_g_ [48,49].

**Figure 5:**
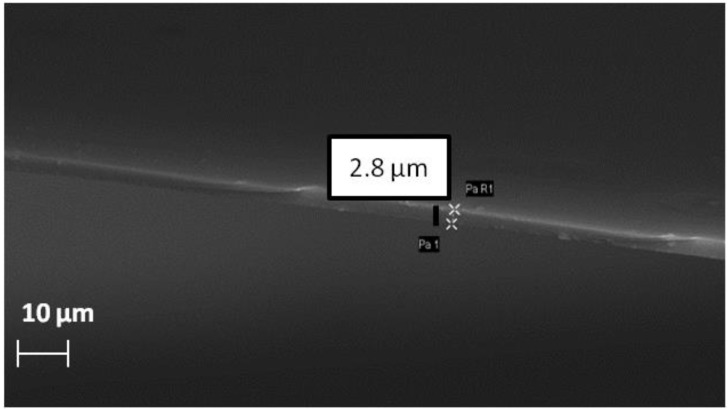
SEM image of PIL containing BF_4_^−^ counter anion.

The membrane morphology and thickness play a crucial role for their potential scale up and commercial use. Although a lot of new materials have been tested as gas separation membranes only very few could have been formed into films thin enough to achieve high fluxes. So far, most of the works report on the use of dense PIL based membranes for CO_2_ gas separation with thickness varying between 50 and 150 μm, while it is generally accepted that it is highly desirable to produce membranes with 0.1–1 μm of thickness in order to achieve much higher flux. In our case, we have successfully prepared by conventional solution casting method very thin, free-standing, homogeneous PIL membranes with a thickness from 3 up to 25 μm. The cross-sectional SEM micrograph of a dense, PIL membrane of just 2.8 μm of thickness is depicted in Figure 5. The excellent film forming ability of the prepared PILs in combination with their high *T*_g_ values make these materials potential candidates to be evaluated as membranes for natural gas purification (CH_4_/CO_2_). 

### 3.2. Gas Separation Properties of Pyridinium Based PILs

Regarding the gas separation properties, the measured single gas permeability values for the precursor membrane (not quaternized polymer) and the corresponding PILs containing BF_4_^−^ and TFSI^−^ as counter anions are given in Table 2. The results show that both PIL membranes containing different anions show improved CO_2_ permeability compared to the precursor. In the case of PIL-TFSI membrane, the CO_2_ and CH_4_ permeabilities are about 3.5 times and 1.5 times higher than those of the precursor membrane, respectively. It appears that both the incorporation of the methyl group in the pyridine ring as well as the inclusion of the fluorinated TFSI anion enable a looser chain packing, thus leading to higher permeabilities. However, in the case of PIL-BF_4_, the CH_4_ permeability is lower in comparison to the one of the precursor polymer. As the BF_4_ anion is less bulky than TFSI, it can be speculated that the BF_4_ anion facilitates polymer packing via inter chain interactions, thus leading to more dense structures and consequently to lower permeabilities. The obtained CO_2_/CH_4_ ideal selectivities of the precursor membranes and the two PIL membranes are also shown in Table 2. It is evident that both PILs (regardless of the counter anion) show improved ideal selectivities compared to their not quaternized precursor membrane.

The effect of the anion on permeability is well documented [15,16] and it was also confirmed by the present measurements. More specifically, the permeability of both CO_2_ and CH_4_ was increased with increasing bulkiness of the anion based on their van der Waal’s increasing volume [50,51] (*V*_w_-BF_4_^−^ = 30.1 Å^3^ < *V*_w_-TFSI^−^ = 88.5 Å^3^). Interestingly, the PIL with the TFSI^−^ anion not only exhibited higher CO_2_ permeability but also maintained comparable ideal selectivity as that of PIL possessing BF_4_^−^ anion. Of course it should be mentioned that these PILs show much lower CO_2_ permeability compared with other rigid, PBI based PILs whereas the CO_2_ permeability values vary in the range of 2–25 Barrer by variation of the polycation substituent or the anion [33]. In the case of PIL based on polyimides containing TFSI as counter ion, CO_2_ permeability up to 28.9 Barrer and CO_2_/CH_4_ ideal selectivity of ~4 were obtained [30]. The low CO_2_ permeabilities of the prepared PIL membranes can be possibly associated with the close polymer chain packing. The incorporation of a small and not bulky group such as methyl probably does not contribute to the significant enhancement of the free volume, which could lead to a more loose polymer chain packing and consequently to higher permeability. The polymer chain packing is related to the degree of quaternization. Recently, A. S. Rewar et al. [32] reported that there is an optimum degree of quaternization (DQ = 13%) of PBI based PILs using a bulky 4-tert-butyl benzyl substituent where the CO_2_ and CH_4_ permeabilities showed maxima, implying the importance of bulky group substitution in retarding chain packing.

Finally, these PILs follow the conventional trade-off relationship, where the PIL-TFSI possessing the highest permeability showed the lowest ideal selectivity for CO_2_ and CH_4_ gases than that of PIL-BF_4_. The obtained CO_2_/CH_4_ ideal selectivities, varying in the range of 41–50, are quite favorable, however permeability has to be further improved as already mentioned.

Mixed gas separation measurements have also been carried out employing a 50/50 CO_2_/CH_4_ mixture (Table 2). The CO_2_ mixed gas permeability of PIL-BF_4_ membrane was found to increase by 45% from 2 to 2.9 Barrer accompanied by an increase in the separation factor to 72 compared to the ideal selectivity of 50. On the other hand, in the case of PIL-TFSI, the CO_2_ mixed gas permeability decreased from 4.1 to 3.4 Barrer while the separation factor (SF) does not change compared to corresponding ideal selectivity. It is evident that in the case of PIL-BF_4_, the mixed gas CO_2_ permeability increases while mixed gas CH_4_ permeability remains almost unchanged. It should be noted that CO_2_ permeability is not constant with the CO_2_ partial pressure and under certain circumstances could be higher in the low CO_2_ pressure range [52]. In the case of PIL-TFSI, the mixed gas permeability of both CO_2_ and CH_4_ appears to decrease to the same extent, which is attributed to a mutual inhibition of permeation for both gases.

### 3.3. Preparation and Characterization of PIL-IL Composite Membranes

Due to the very low CO_2_ permeabilities obtained, an additional direction was to prepare PIL-IL composite membranes as a means to boost CO_2_ permeability. This is a common strategy adopted by various groups since the incorporation of free IL generally leads to a significant increase of the gas permeabilities of the PIL based membranes [23,53,54]. PIL-IL composite membranes are blends that combine the permeation properties of the PIL with IL, where ILs have the ability to strongly interact with the charged backbone of the PILs through electrostatic interactions, thus leading to the free volume increase, and enhancement of the polymer chain mobility and consequently to CO_2_ permeability increase. Moreover, these strong electrostatic forces hold the IL into the PIL polymer matrix, thus preventing the leaching under high transmembrane pressure differentials.

Different amounts (30, 40, 45 wt %) of [bmpy][TFSI]-IL1 were blended with PILs containing the TFSI^−^ anion (in both IL and PIL) to evaluate the effect of the free IL on the physicochemical and gas transport properties (Table 1 and Scheme 2).

**Figure 6:**
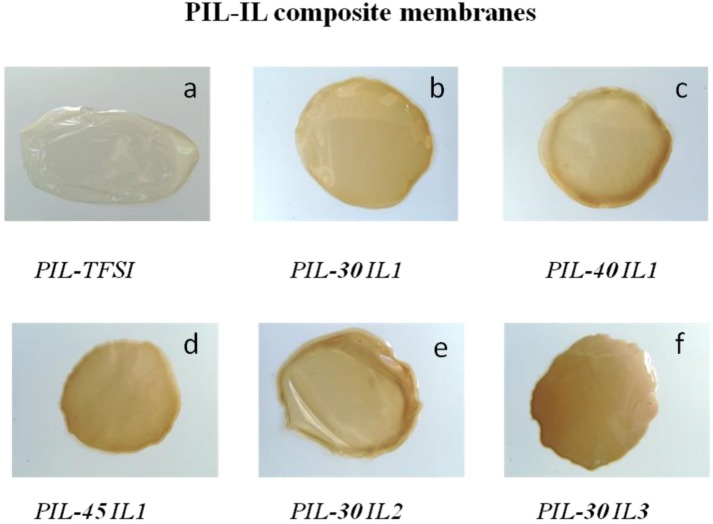
Images of the prepared (**a**) PIL-TFSI and PIL-IL composite membranes with (**b**) 30 wt % of IL1, (**c**) 40 wt % of IL1, (**d**) 45 wt % of IL1, (**e**) 30 wt % of IL2 and (**f**) 30 wt % of IL3.

In this case, the [bmpy][TFSI] was chosen due to its structural similarity with the pyridinium unit of the PIL. Homogeneous, free-standing PIL-IL1 composite membranes using the solution casting method have been obtained in all cases (Figure 6).

**Figure 7:**
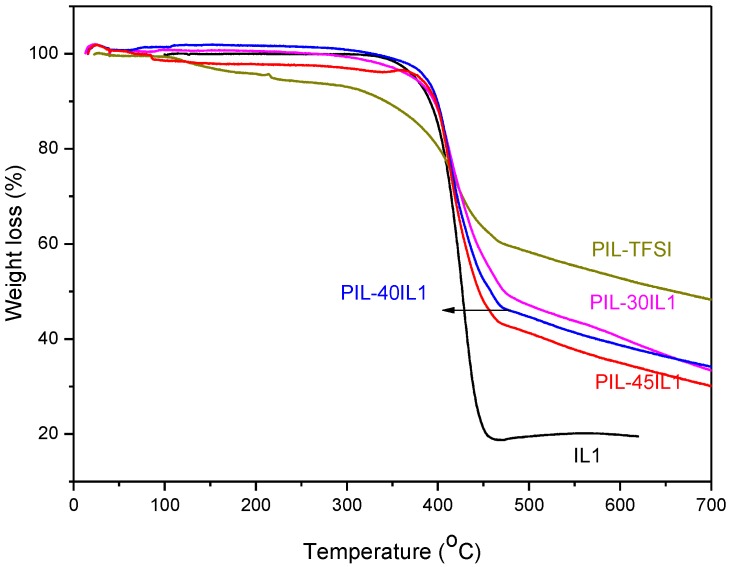
TGA curves of neat PIL and PIL-IL1 composite membranes.

The thermal stability of the synthesized PIL and the PIL-IL1 composite membranes was assessed by TGA analysis (Figure 7). The effect of the IL content on the thermal stability of the prepared PIL-IL1 composite membranes was studied. It is evident that the addition of free IL1 increases the thermal stability of the PIL-IL1 composite membranes with respect to the pure PIL. The improved thermal behavior of the PIL-IL membranes compared to the neat PIL could be due to strong electrostatic interactions developed between the cationic polymer backbone and the free IL. The onset of thermal degradation for the three PIL-IL1 composite membranes appeared between 200 and 270 °C.

**Figure 8:**
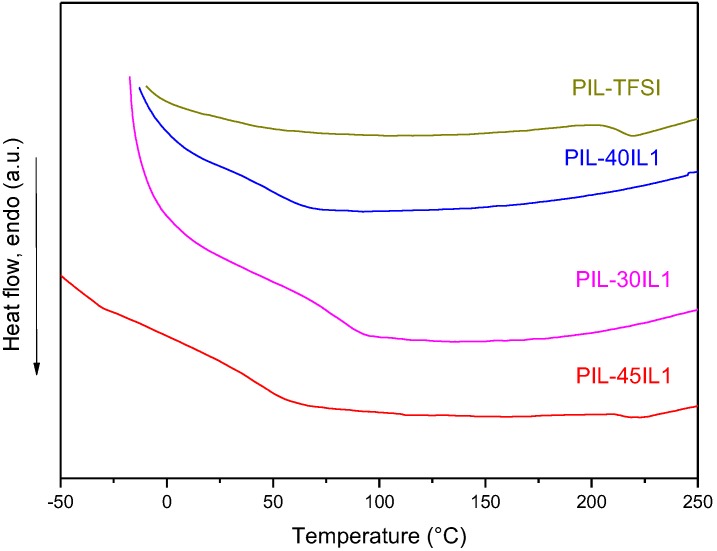
DSC curves of neat PIL and PIL-IL1 composite membranes.

The thermal properties were further studied by DSC, as depicted in Figure 8. A substantial decrease in the *T*_g_ was observed upon increasing percentage of the free IL1 contained in the PIL-IL1 composite membranes. In specific, the *T*_g_ of neat PIL containing TFSI^−^ as counter anion is 214 °C while the pure free IL1 shows a *T*_g_ at −75 °C which is close with the *T*_g_ value (−80 °C) observed by Papaiconomou et al. [55]. The *T*_g_s of PIL-IL1 composite membranes with 40 and 45 wt % of IL1 are 54 and 49 °C, respectively.

### 3.4. Gas Separation Properties of PIL-IL Composite Membranes

**Figure 9:**
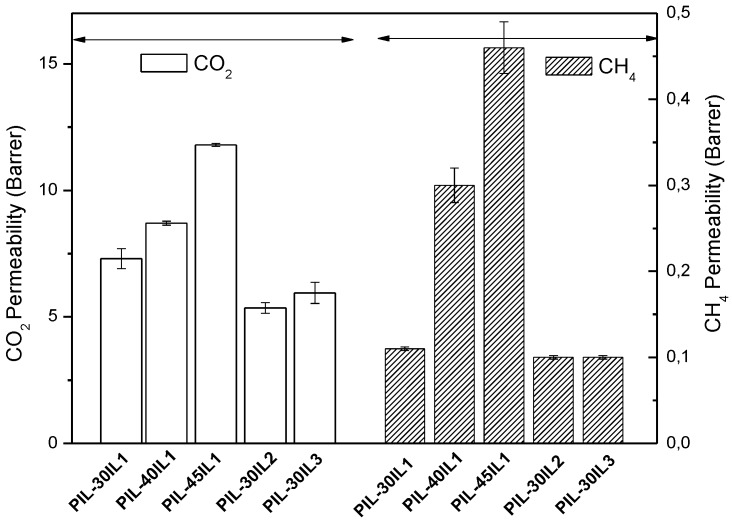
CO_2_ and CH_4_ single gas permeabilities of the prepared PIL-IL composite membranes at room temperature.

Regarding the study of the gas separation properties of PIL-IL1 composite membranes, it was observed that both CO_2_ and CH_4_ single gas permeabilities increased by increasing the amount of free IL incorporated in the polymer matrix, as illustrated in Figure 9. The permeability enhancement with increasing the IL1 percentage is related to the increase of the free volume of the PIL-IL composite membranes resulting in higher polymer chain mobility due to the presence of free IL ion pairs. More specifically, the incorporation of free ILs into the PIL matrix, where the ILs serve as a plasticizer results in gas diffusivity increase as reported by Bara et al. [53]. Since CO_2_ diffuses much faster in the IL phase than in the solid PIL phase, it can be assumed that most of the CO_2_ permeability increase is governed by the amount of free IL in the PIL-IL composite membranes [56]. This behavior was also observed for other PIL-IL composite membranes [28,34,57,58,59,60,61,62]. The composite membrane with 55/45 composition showed the highest CO_2_ single gas permeability (11.8 Barrer) and high CO_2_/CH_4_ ideal selectivity (α CO_2_/CH_4_ = 35), as shown in Table 3. In comparison to pure PIL-TFSI, the former exhibits 3 times higher CO_2_ single gas permeability and comparable ideal selectivity.

In addition, composite membranes bearing the same PIL-TFSI and different IL cations containing the TFSI^−^ anion (IL2 and IL3 containing hexyl and dodecyl imidazolium, respectively) were also prepared to study the influence of the IL cation and IL cation substitution on the gas separation properties (Table 1). In fact, homogeneous, free standing membranes with 30% of [c4mim][TFSI]-IL2 (PIL- 30 IL2 membrane) and 30% of [c12mim][TFSI]-IL3 (PIL- 30 IL3 membrane) have been successfully obtained (Figure 6).

In what concerns the IL cation effect, PIL-IL2 membrane with composition 70/30 was compared with the corresponding PIL-IL1 with the same composition. The membrane prepared with IL1 exhibited improved CO_2_ and CH_4_ single gas permeabilities than its analogue prepared with IL2 probably due to similar structures of PIL and IL1 (Figure 9 and Table 3).

The study of the effect of the alkyl substituent of imidazolium-based free ILs (IL-2 and IL-3 are composed of hexyl and dodecyl imidazolium-based IL, respectively) on the gas separation properties was made by comparing the PIL-IL2 and PIL-IL3 composite membranes with composition 70/30. It was shown that the alkyl substituent has almost no effect on CO_2_ and CH_4_ single gas permeabilities as well as on CO_2_/CH_4_ ideal selectivities (Figure 9 and Table 3).

Regarding the mixed gas separation measurements for PIL-IL1 composite membranes, it is generally observed that the separation factor for PIL-IL membranes containing 30 and 40 wt % of IL1 is smaller than the corresponding ideal selectivity while the CO_2_ mixed gas permeability increases slightly (Table 3). In the case of PIL-45IL1 (45 wt % IL1), the CO_2_ mixed gas permeability remains almost unchanged compared to the corresponding CO_2_ single gas case. The presence of IL1 appears to facilitate the permeation of both gases with CH_4_ being slightly favored over CO_2_. To the best of our knowledge, it should be noted that there are no literature data concerning mixed gas permeation of the employed pyridinium type IL and, as a result, no comparison can be made with previous reports.

**Figure 10:**
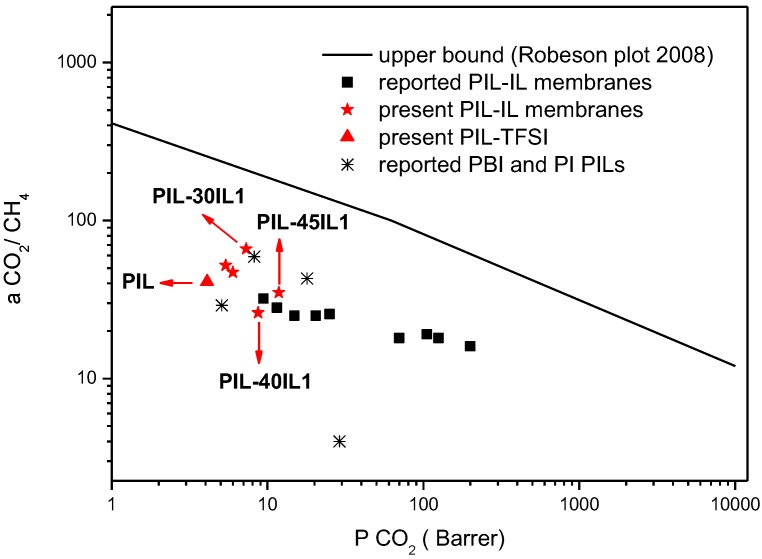
CO_2_/CH_4_ separation performance of the prepared membranes. Data are plotted on a log-log scale and the upper bound line is adapted from the respective Robeson plot [20]. Literature data reported for other PIL-IL composite membranes (∎) [30,60,61,62] and PIL membranes (✳) [30,33].

Comparison of the data of this work regarding the CO_2_/CH_4_ separation performance with those reported in the literature for other PIL-IL as well as PIL membranes comprising the TFSI anion are illustrated in a Robeson plot [20] (Figure 10). This plot is used to benchmark the gas separation performance of different membrane materials and the “upper bound” line has been adapted from the corresponding figure in Ref. [20]. The Robeson plot in Figure 10 indicates that the prepared PIL-IL membranes lie below the upper bound limit on the left part of the plot characterized by lower permeabilities and higher selectivities compared to other reported PIL-IL membranes. The prepared PIL membranes are placed northwest of other rigid PILs reported in the literature (e.g., PBI based and PI based PILs). Evidently, progress should be made in improving the permeability of the family of membranes studied in the present work. However, it should be mentioned that the novel aspect of the present work lies in the new PIL structure that was successfully synthesized, and has led to robust, very thin membranes down to 3 μm thickness. This PIL was also served as a matrix for the incorporation of high amounts of free ionic liquids resulting still in mechanically stable PIL-IL membranes with improved CO_2_ permeability. At the same time, although the specific PIL and its respective PIL-IL blend membranes do not lead to sufficiently high CO_2_ permeability for practical applications, nevertheless, the detailed study of their gas transport properties is important to understand how the PIL structure affects the gas permeability and to provide useful guidelines for further designing new PILs and PIL-IL membranes with improved permeability and stability.

The design and synthesis of new, cross-linked PIL-IL membranes that combine the high IL loadings, which can boost CO_2_ permeability with the increased stability via the formation of a network that prevents the leaching of the IL, is a topic that we are currently working in order to further develop novel polymeric structures with the desirable transport properties and stability.

## 4. Conclusions

Novel pyridinium based PILs have been synthesized through conversion of high molecular weight aromatic polyethers bearing main chain pyridine groups to their ionic analogues via N-methylation reaction, followed by anion exchange methathesis reaction. The prepared PILs show high *T*_g_ values, high thermal stability and have the ability to form very thin, free standing films with thickness ranging from 3 to 25 μm via a conventional solution casting method. PIL containing the TFSI anion showed higher CO_2_ and CH_4_ permeability compared to its analogue containing the BF_4_ anion, denoting the important role of anion in gas transport properties. PIL-IL composite membranes containing the TFSI^−^ anion (in both IL and PIL) were also prepared by blending PIL with different amounts (30, 40, 45 wt %) of a pyridinium based IL. The addition of the IL into the PIL matrix resulted in the enhancement of both CO_2_ and CH_4_ permeabilities of the PIL-IL composite membranes compared to the neat PIL. Moreover, both CO_2_ and CH_4_ permeabilities of the PIL-IL composite membranes were increased by increasing the IL content. Composite membranes comprised of PILs and different imidazolium-based IL cations were fabricated and it was shown that PIL-IL membrane containing the pyridinium based IL showed improved CO_2_ and CH_4_ permeabilities compared to its analogues containing the imidazolium-based ILs sharing the same composition, probably due to the structural similarity.

Although, it is evident from the obtained results that the prepared pyridinium based PILs and their corresponding PIL/IL composite membranes cannot satisfy the high permeabilities required for e.g., CO_2_/CH_4_ separation, however, the knowledge gained through the new PIL and PIL-IL membranes developed could be used for the preparation of ultra thin film composite membranes targeting to much higher CO_2_ permeance.

## Figures and Tables

**Table 1:** Composition of the prepared PIL-IL composite membranes (PIL refers to the PIL containing TFSI as counter anion).

Composite Membranes	IL	IL wt %	Solvent
PIL-30 IL1	IL1-[bmpy][TFSI]	30	DMAc
PIL-40 IL1	IL1-[bmpy][TFSI]	40	DMAc
PIL-45IL1	IL1-[bmpy][TFSI]	45	DMAc
PIL-30 IL2	IL2-[c6mim][TFSI]	30	DMAc
PIL-30 IL3	IL3-[c12mim][TFSI]	30	DMAc

**Table 2:** Separation performance of the prepared polymer precursor Py-APE, PIL-BF_4_ and PIL-TFSI membranes at room temperature (1 Barrer $=\;10^{-10}\;{\mathrm{cm}}^{3}{(\mathrm{STP})\;\mathrm{cm}\;\mathrm{cm}}^{-2}{\;s}^{-1}{\;\mathrm{cm}\;\mathrm{Hg}}^{-1}$).

Polymeric Membrane	Single Gas Permeability		Ideal Selectivity	Mixed Gas Permeability	Mixed Gas Selectivity
Compound	CO_2_ (Barrer)	CH_4_ (Barrer)	α CO_2_/CH_4_	CO_2_ (Barrer)	SF CO_2_/CH_4_
Py-APE	1.2 ± 0.1	0.07 ± 0.01	17	-	-
PIL-BF_4_	2.0 ± 0.2	0.04 ± 0.01	50	2.9 ± 0.1	72
PIL-TFSI	4.1 ± 0.1	0.10 ± 0.01	41	3.4 ± 0.1	40

**Table 3:** Separation performance of the prepared PIL-TFSI and PIL-IL membranes at room temperature (1 Barrer $=\;10^{-10}\;{\mathrm{cm}}^{3}{(\mathrm{STP})\;\mathrm{cm}\;\mathrm{cm}}^{-2}{\;s}^{-1}{\;\mathrm{cm}\;\mathrm{Hg}}^{-1}$).

Compound	CO_2_ Single Gas Permeability (Barrer)	α CO_2_/CH_4_ (Ideal Selectivity)	CO_2_ Mixed Gas Permeability (Barrer)	SF CO_2_/CH_4_ (Mixed Gas Selectivity)
PIL-TFSI	4.1 ± 0.1	41	3.4 ± 0.1	40
PIL-30IL1	7.3 ± 0.4	66	9.0 ± 0.2	60
PIL-40IL1	8.7 ± 0.1	26	10.0 ± 0.2	25
PIL-45IL1	11.8 ± 0.1	35	11.1 ± 0.2	26
PIL-30IL2	5.4 ± 0.2	52	-	-
PIL-30IL3	6.0 ± 0.4	47	-	-

## Supplementary Materials

The following are available online at http://www.mdpi.com/2073-4360/10/8/912/s1, Figure S1: ^19^FNMR spectrum for PIL containing TFSI^−^ as anion, Figure S2: ^19^FNMR spectrum for PIL containing BF_4_^−^ as anion.
