# Supplementary material for: New Pyridinium Type Poly(Ionic Liquids) as Membranes for CO2 Separation
Source: Polymers (Basel). 2018 Aug 13;10(8):912. doi: 10.3390/polym10080912 (PMC6403706; doi:10.3390/polym10080912)
Supplement: Supplementary file 1 [file polymers-10-00912-s001.pdf]

Supporting Information

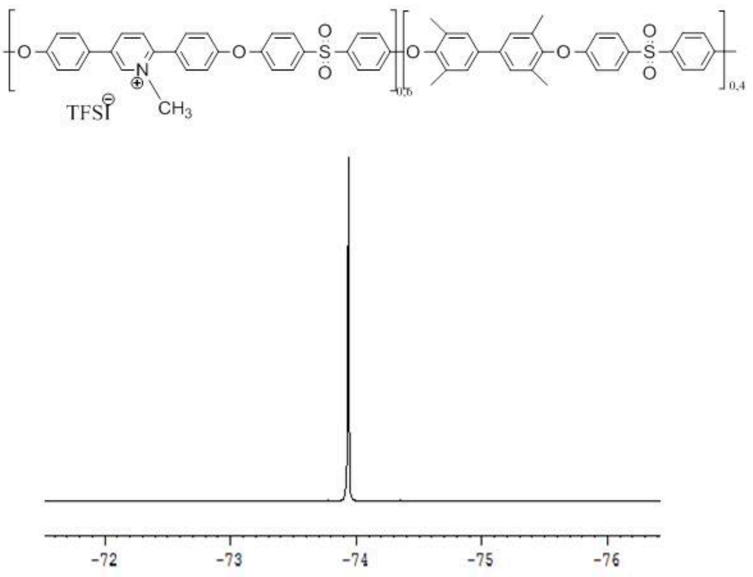

Figure S1. ^{19}FNMR spectrum for PIL containing $TFSI^-$ as counter anion.

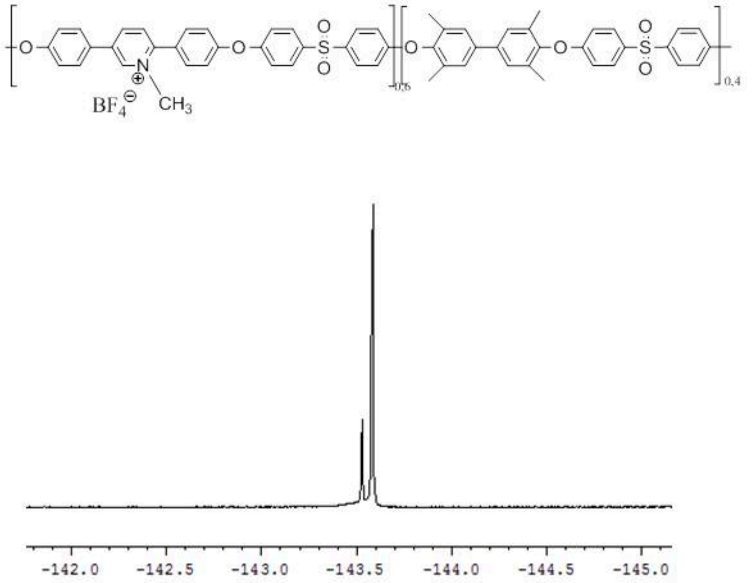

Figure S2. ^{19}FNMR spectrum for PIL containing BF_4^- as counter anion.
